# The potential of ferroptosis combined with radiotherapy in cancer treatment

**DOI:** 10.3389/fonc.2023.1085581

**Published:** 2023-03-17

**Authors:** Zekun Lu, Bingkai Xiao, Weibo Chen, Tianyu Tang, Qifeng Zhuo, Xuemin Chen

**Affiliations:** ^1^ Department of Hepatopancreatobiliary Surgery, the Third Affiliated Hospital of Soochow University, Changzhou, Jiangsu, China; ^2^ Department of Hepatabiliary Surgery, The Second People’s Hospital of Changshu, The Affiliated Changshu Hospital of Xuzhou Medical University, Changshu, China; ^3^ Department of Pancreatic Surgery, Fudan University Shanghai Cancer Center, Shanghai, China

**Keywords:** ferroptosis, radiotherapy, cancer treatment, drug resistance, signaling pathways

## Abstract

Ferroptosis is a new form of regulatory cell death that is closely related to the balance of redox reactions and the occurrence and development of cancer. There is increasing evidence that inducing ferroptosis in cells has great potential in the treatment of cancer. Especially when combined with traditional therapy, it can improve the sensitivity of cancer cells to traditional therapy and overcome the drug resistance of cancer cells. This paper reviews the signaling pathways regulating ferroptosis and the great potential of ferroptosis and radiotherapy (RT) in cancer treatment and emphasizes the unique therapeutic effects of ferroptosis combined with RT on cancer cells, such as synergy, sensitization and reversal of drug resistance, providing a new direction for cancer treatment. Finally, the challenges and research directions for this joint strategy are discussed.

## Introduction

Ferroptosis is an iron-dependent form of regulated cell death that, unlike other forms of cell death (such as apoptosis and necrosis), destroys the integrity of cell membranes but does not cause cell swelling; it is not dependent on caspase activation and is not inhibited by apoptosis inhibitors. The main characteristics of ferroptosis are the accumulation of iron ions and lipid peroxide, decreased numbers of mitochondria and reduced numbers or disappearance of mitochondrial cristae. Induction of apoptosis was previously considered to be the main pathway leading to cancer cell death during cancer treatment, but an increasing number of studies have shown that inducing cancer cells to undergo ferroptosis by regulating key molecules in the ferroptosis signaling pathway not only can improve the ability to kill cancer cells but also provides a new therapeutic strategy for refractory cancers. RT kills tumor cells by damaging DNA with radiation, or by destroying biomolecules with free radicals (mainly ROS). As the field of RT continues to advance, RT can achieve better local control and improved clinical outcomes. At the same time, the new understanding of radiobiology has enabled the development of radiation therapy as a personalized cancer treatment. RT can also act as a vaccine by altering the tumor microenvironment to enhance the immune system’s ability to recognize tumor cells. The key problem with RT in cancer treatment is that there can be radiotherapy resistance which makes radiotherapy less effective in cancer treatment. The combination of ferroptosis and RT in cancer also improves the ability to kill cancer cells in addition to reversing the RT resistance of cancer cells, providing a new method for cancer treatment. This article reviews the signaling pathways regulating ferroptosis, the great potential of ferroptosis for treating cancer, the role and advances in the understanding of RT in cancer and the unique effects of sensitization and synergy induced by the combination of ferroptosis and RT in cancer. We also discuss the challenges faced by this combination therapy and future research directions

## The discovery of ferroptosis and signaling pathway

Ferroptosis is a kind of regulatory cell death characterized by iron overload, lipid reactive oxygen accumulation and lipid peroxidation. In 2003, a new compound named erastin was discovered. It has a lethal effect on human foreskin fibroblasts expressing RAS but has no killing effect on homologous primary cells (1). In 2008, Ras-selective lethal compounds (RSLs), specifically, RSL3 and RSL5, were also found to selectively kill cancer cells expressing the RAS gene *via* a nonapoptotic mechanism (2). Moreover, necrostatin-1 and wortmannin inhibitors can inhibit cell necrosis, apoptosis and autophagy but cannot block the cell death induced by RSLs (3). In contrast, the iron chelator deferoxamine mesylate and the antioxidant vitamin E can inhibit the cell death induced by RSLs (3). In 2012, Dixon et al. officially identified and named ferroptosis according to the characteristics of the mechanism by which erastin kills cancer cells through RAS mutation (3). Ferroptosis was identified as a new form of cell death. The morphological, biochemical and genetic characteristics of ferroptosis are also different from those of necrosis, apoptosis, autophagy and other regulatory forms of cell deaths (4, 5). Morphologically, ferroptosis mainly occurs in cells, showing a decrease in mitochondrial volume, a decrease or disappearance of mitochondrial cristae and an increase in membrane density (2, 3). However, the size of the nucleus is normal, and the cell membrane is intact. In terms of biochemistry, the activities of glutathione peroxidase 4 (GPx4) and intracellular glutathione (GSH) are decreased. Lipid peroxide cannot be reduced to the corresponding aliphatic alcohols by the reduction reaction catalyzed by GPx4. Fe2+ oxidizes lipids in a Fenton-like manner, producing a large amount of ROS and promoting ferroptosis (2, 6, 7). In terms of genes, ferroptosis is a biological process regulated by multiple genes. Ferroptosis is mainly related to genetic changes in lipid peroxidation and iron metabolism. In recent years, ferroptosis has attracted increasing attention. As it is a new form of regulatory cell death, the discovery of ferroptosis provides new ideas for the understanding and treatment of many diseases, especially cancers.

Ferroptosis is mainly caused by the formation of iron-dependent lipid peroxides, which is regulated by many factors. Next, the signaling pathways regulating this process are introduced.

### The system XC−/GSH/GPx4 axis

**Figure 1:**
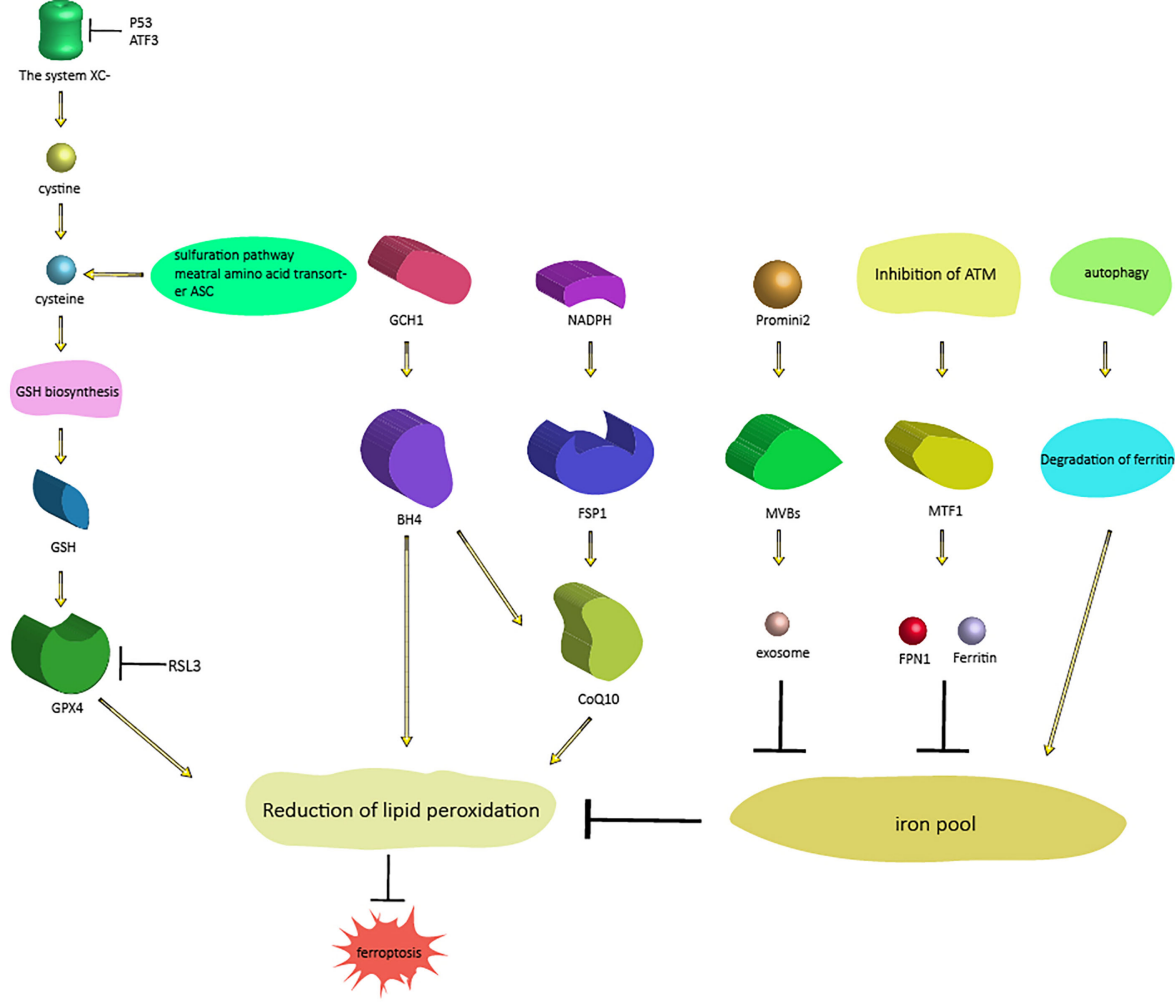
Signaling pathways regulating ferroptosis. There are two different processes that regulate ferroptosis. One process includes three pathways that regulate ferroptosis (from right to left) by regulating intracellular iron content: the autophagy pathway, the ferritin-FPN1 pathway [ATM-MTF1-ferritin-FPN1], and the exosome pathway [Prominin2-MVB-exosomes]; the other process regulates another three pathways of ferroptosis (from left to right) by regulating lipid peroxidation: the GSH pathway, BH4 pathway [GCH1-BH4] and CoQ10 pathway [NAD(P)H-FSP1-CoQ10]. As mentioned above, these proteins and genes are also affected by upstream proteins and genes. Abbreviations: MTF1, metal-regulated transcription factor 1; FPN1, ferroportin 1; MVBs, multivesicular bodies; GSH, glutathione; GPX, glutathione peroxidase; GCH1, GTP cyclohydrolase-1; BH4, tetrahydrobiopterin; FSP1, ferroptosis suppressor protein 1.

Lipid peroxides, which cause ferroptosis, can be endogenously counteracted by the system XC-/GSH/GPx4 axis (3, 7, 8); therefore, interfering with any step of this signaling pathway may cause ferroptosis. The most upstream component of the axis is system XC-, a highly specific cystine uptake system that transports cystine (the oxidized form of cysteine) into cells and transports glutamate out of cells at a 1:1 ratio (9). System XC- is composed of a light chain encoded by solute carrier family 7 member (SLC7A11) and a heavy chain (4f2hc), belonging to the heterodimer amino acid transporter family. The activity of system XC- is usually positively correlated with the expression level of the light chain, and the light chain is regulated by complex transcription (9–11).Cystine enters cells through system XC- and is reduced to cysteine by GSH or thioredoxin reductase 1, which is then used for GSH biosynthesis (12). Although this route is the most relevant way to address the demand for cysteine in cell culture (almost all cysteine is oxidized to cystine), cysteine can also be provided to some extent by the sulfuration pathway and the neutral amino acid transporter ASC (10). The latter is likely to be the most important mode in the whole organism, where the majority of plasma and tissue cysteine is present in the reduced form. Cysteine is the rate-limiting substrate for glutathione biosynthesis, and GSH is the strongest antioxidant in cells; glutathione-S-transferase (GST) and glutathione peroxidase (GPX) catalyze the detoxification of various electrophilic compounds and peroxides. Therefore, blocking the level of intracellular cysteine can affect the level of GSH, which makes cells vulnerable to oxidative damage (13). GPX is a highly conserved enzyme in evolution. It uses GSH as a cofactor to reduce peroxide to corresponding alcohols, thus limiting the formation of transition metal-dependent toxic free radicals. According to amino acid sequence similarity, the eight mammalian GPX proteins can be divided into three categories: GPx1 and GPx2; GPx3, GPx5 and GPx6; and GPx4, GPx7 and GPx8 (14). GPx1-4 and GPx6 (in humans) are selenoproteins, which contain essential selenocysteine in the active site of the enzyme, whereas GPx5, GPx 6 (in mice and rats), GPx7 and GPx8 use the active site cysteine. In contrast to other family members, GPx4 (PHGPx) can be used as a phospholipid catalase to reduce lipid peroxides to aliphatic alcohols (15, 16). Therefore, the activity of GPx4 can maintain the dynamic balance of lipids in cells, prevent the accumulation of toxic lipid reactive oxygen species (ROS) and prevent ferroptosis. In conclusion, system XC-, GSH and Gpx4 together constitute the signaling pathway (**Figure 1**) regulating ferroptosis; any substance that affects the availability of cysteine, the biosynthesis of GSH and the normal function of GPx4 will cause the accumulation of lipid peroxide and further lead to ferroptosis. For example, p53 reduces system XC- activity by inhibiting SLC7A11 expression, leading to ferroptosis (17). Erastin can prevent extracellular cystine from entering cells by inhibiting system XC-, thus reducing intracellular GSH levels. GSH is an indispensable substrate for the antioxidant activity of GPx4. Therefore, if the activity of GPx4 is decreased, the redox dynamic equilibrium is destroyed, and intracellular ROS accumulate, leading to ferroptosis (18). Activating transcription factor 3 (ATF3) inhibits system XC- by binding to the SLC7A11 promoter and inhibiting SLC7A11 expression in a p53-independent manner, thus reducing intracellular GSH levels, promoting erastin-induced lipid peroxidation and eventually leading to ferroptosis (11). RSL3 causes lipid peroxide accumulation by inhibiting the activity of GPx4 and ultimately leads to ferroptosis (19).

### The NAD(P)H-FSP1-CoQ10 axis

CoQ10 is not only an important part of the mitochondrial electron transport chain but also inhibits lipid peroxidation by capturing free radical intermediates outside mitochondria. Therefore, the absence of CoQ10 makes cells sensitive to ferroptosis (20). In 2019, Bersuker et al. identified ferroptosis suppressor protein 1 (FSP1) (formerly known as apoptosis-inducing factor mitochondrial 2 (aifm2)) as an effective ferroptosis resistance factor by using synthetic lethal CRISPR/cas9 screening (21). FSP1 prevents lipid peroxidation and inhibits ferroptosis by using NAD(P)H to regenerate reduced CoQ10, which does not require GPx4 or GSH (21, 22). This finding revealed a new NAD(P)H-FSP1-CoQ10 signaling pathway (**Figure 1**), which acts in parallel with GSH-GPX4 to inhibit ferroptosis.

### The GCH1-BH4-phospholipid axis

In 2019, Kraft et al. identified a group of genes that antagonize ferroptosis through genome-wide activation screening, including GTP cyclohydrolase-1 (GCH1) and its metabolic derivative tetrahydrobiopterin/dihydrobiopterin (BH4/BH2) (23). GTP cyclohydrolase, encoded by the GCH1 gene, is the key enzyme in the *de novo* synthesis of BH4; BH4 is an important cofactor of nitric oxide synthase, and BH4 can also aid the formation of reduced CoQ10 (20); therefore, an insufficient supply of BH4 will lead to the uncoupling of nitric oxide synthase and the production of hyperoxia free radicals (24). GCH1-expressing cells induce lipid remodeling by synthesizing BH4/BH2, and inhibit ferroptosis by selectively preventing two polyunsaturated fatty acyl tails from consuming phospholipids (23).

Therefore, the GCH1-BH4-phospholipid axis (**Figure 1**) is the main regulator of ferroptosis resistance and controls the production of endogenous antioxidant BH4, the abundance of CoQ10, and the peroxidation of abnormal phospholipids and two polyunsaturated fatty acid (PUFA) acyl tails (23). This finding reveals a signaling pathway regulating ferroptosis independent of GPx4 and GSH.

### Autophagy and ferroptosis

The term “autophagy” was proposed by Christian de Duve in 1963. It is used to describe the process of degradation of intracellular components by lysosomes (25).Autophagy controls cell survival and death by regulating the quantity and quality of proteins and organelles (26). The main autophagy pathway is composed of more than 30 autophagy-related proteins, including LC3B and BECN1. LC3B is a structural component of the autophagy pathway (27), and BECN1 is a promoter of autophagy. In the process of autophagy, double-layered membrane vesicles containing denatured and necrotic organelles and proteins are formed in the cytoplasm, the outer membrane of the autophagosome fuses with the lysosome, and the inner membrane and its substances enter the lysosome cavity to form autolysosomes. Under low pH conditions, lysosomal hydrolase acts on the autolysosome, thus degrading these substances (28). An increase in autophagic flux in different cells is observed under the action of classical ferroptosis activators such as RSL3 and erastin. Autophagy plays an important role in the induction of ferroptosis by regulating iron homeostasis and ROS production (29, 30). In 2019, Park et al. confirmed that autophagy was indeed induced by ROS induced by the ferroptosis inducer(FIN) erastin and found that autophagy led to iron-dependent ferroptosis through degradation of ferritin and induction of transferrin receptor 1 (TfR1) expression; in the process of ferroptosis induced by erastin, autophagy defects led to iron depletion and lipid peroxidation reduction, thus leading to cell survival (31). Ferritin is the main intracellular protein that stores iron. Active iron (Fe2+) causes toxic Fenton-type oxidation, while iron in the nonreactive state (Fe3+) stored in ferritin is less harmful. when ferroptosis is induced by molecules such as erastin, autophagy promotes the degradation of ferritin, which results in the release of the chelated iron in ferritin, which leads to an increase in the intracellular unstable iron pool and subsequent oxidative stress, ultimately leading to the occurrence of ferroptosis (32). Therefore, autophagy controls ferroptosis (**Figure 1**) by regulating ferritin degradation and TfR1 expression.

### ATM-MTF1-Ferritin/FPN1 axis

ATM is a serine/threonine kinase in the phosphatidylinositol 3-kinase-like kinase family (PIKK). Metal-regulated transcription factor 1 (MTF1) is a highly conserved metal-binding transcription factor in eukaryotes that binds to conserved DNA sequence motifs called metal response elements (MREs) to promote gene transcription that maintains metal homeostasis. Normally, MTF1 is mainly localized to the cytoplasm; when MTF1 is activated, it moves from the cytoplasm to the nucleus, where it recognizes and interacts with the MREs of genes that regulate homeostasis. MTF1 responds to both excess metal and metal deficiency to protect cells from oxidative and hypoxic damage (33). Ferritin is an intracellular protein that stores intracellular free iron in a nontoxic and bioavailable form. Ferroportin 1 (FPN1), the only known iron export protein on cell membranes, is encoded by the FPN1 (SLC40A1) gene and transfers iron from the external environment (such as the intestinal lumen) and from the interior iron stores to the blood (34).Inhibition of ATM enhances the nuclear translocation of MTF1, which regulates the expression of ferritin/FPN1, thereby increasing the expression of iron-regulated factors related to iron storage (ferritin heavy and light chains, FTH-1 and FTL) and export (FPN1), thus protecting cells from the threat of ferroptosis. In the process of inhibiting ATM, synergistic changes in these iron regulators lead to a decrease in active iron, thereby preventing iron-dependent ferroptosis (35). These mechanisms reveal that the ATM-MTF1-ferritin/FPN1 axis is a novel signaling pathway regulating ferroptosis (**Figure 1**). Prominin2 also regulates ferroptosis by regulating intracellular iron content.Prominin2, a pentagonal protein involved in lipid dynamics regulation, prevents ferroptosis(**Figure 1**) by promoting the formation of ferritin-containing multivesicular bodies (MVBs) and exosomes that then transport iron out of cells. These results also reveal a signaling pathway that regulates ferroptosis driven by the Prominin2-MVB-exosome-ferritin pathway (36).

### NRf2 and PI3K-AKT-mTOR axis

Nuclear factor erythroid 2-related factor 2 (NRf2) regulates ferroptosis in various ways. When cells are exposed to ferroptosis inducers, the SQSTM1/p62-Keap1-NRf2-AKR1C (metal-binding protein MT-1G) pathway is activated, which then activates the transcription of quinone oxidoreductase-1, HO-1, and ferritin heavy chain-1, ultimately reducing sensitivity to ferroptosis (37). NRf2 can also directly or indirectly regulate the expression and function of GPX4 to regulate the sensitivity of cancer cells to ferroptosis (38). NRf2 directly binds to the ARE sequence of the SLC7A11 subunit promoter and then promotes SLC7A111 expression (39). NRf2 overexpression or Keap1 knockdown increases SLC7A11 expression, whereas inhibition of NRf2 expression or Keapp1 overexpression reduces SLC7A111 protein expression, thereby altering sensitivity to ferroptosis (40, 41). When Keap1 is inhibited, NRf2 activity increases, leading to upregulation of the ATP-binding cassette (ABC) -family transporter multidrug resistance protein (MRP1), which prevents GSH efflux from the cell and strongly inhibits iron ptosis (42). Thus, NRf2 may regulate ferroptosis by partially targeting SLC7A11 to regulate GPX4 synthesis and function (43).

Alterations in the phosphatidylinositol 3-kinase-Serine/threonine kinase AKT (PI3K-AKT) signaling pathway are associated with the progression of multiple cancers. mTOR is a downstream participant in the PI3K-AKT signaling pathway, which regulates tumor cell function. PI3K mutation imparts resistance to ferric sagging to cancer cells, and inhibition of PI3K-AKT-mTOR signaling axis can sensitise cancer cells to ferriptosis (44). Mechanistically, this resistance to ferriptosis requires sustained activation of mTORC1 and its dependent mechanistic target of sterol regulatory element binding Protein 1 (SREBP1), a central transcription factor that regulates lipid metabolism. At the same time, stearoyl-coA desaturase-1 (SCD1) is the transcriptional target of SREBP1, and inhibits the activity of SREBP1 by producing monounsaturated fatty acids. Thus PI3K-AKT-mTOR pathway activation inhibits ferriptosis through SREBP1/SCD1-mediated lipogenesis (45).

### Ferroptosis and RT

#### The role of ferroptosis in cancer

During tumor development, cancer cells can undergo several forms of regulated cell death, including apoptosis, autophagy and necrosis (46). In addition, to promote growth and development, cancer cells show higher iron demand than normal cells; this dependence on iron makes cancer cells more vulnerable to iron catalyzed death, which is called ferroptosis (47). Therefore, ferroptosis also plays a role in the development of cancer. Increasing evidence has shown that ferroptosis plays an important role in tumor inhibition. Specifically, knockout of GPx4 by siRNAs reduces the level of GPx4 protein (GPx4 protein is the central mediator of ferroptosis) and then leads to the death of renal cell carcinoma cells, accompanied by the production of lipid ROS (8, 46). This process can be blocked by the iron chelator and antioxidant vitamin E. In addition, inhibition of SLC7A11, a member of the cystine/glutamate antiporter, can cause ferroptosis (3). SLC7A11 is highly expressed in human tumors (48, 49), and its high expression can prevent ferroptosis in cancer cells. These findings suggest that ferroptosis is a key factor controlling the development of cancer. It has also been found that ferroptosis can inhibit the proliferation of malignant cells in pancreatic cancer, liver cancer, breast cancer, prostate cancer and other cancers (50–53); specifically, some highly malignant cancer cells have been proven to have congenital susceptibility to ferroptosis, so ferroptosis induction could represent a new cancer treatment stratey (18).

Tumor cells evade other forms of cell death by maintaining or gaining sensitivity to ferroptosis; therefore, ferroptosis can be induced to treat refractory tumors (54). For example, mutation of three lysine residues in the DNA-binding domain of mutant p53 (p533KR), which makes the residues unable to be acetylated, is sufficient to inhibit tumor growth in mice, although the mutant’s ability to induce apoptosis, cell cycle arrest and senescence is weakened (54, 55). However, it is worth noting that the p533KR mutant can still inhibit system XC- transcription by directly binding to the promoter, so the cells are sensitive to ROS-induced ferroptosis (56). Therefore, p53 can inhibit the occurrence and development of refractory tumors by inducing ferroptosis.

**Figure 2:**
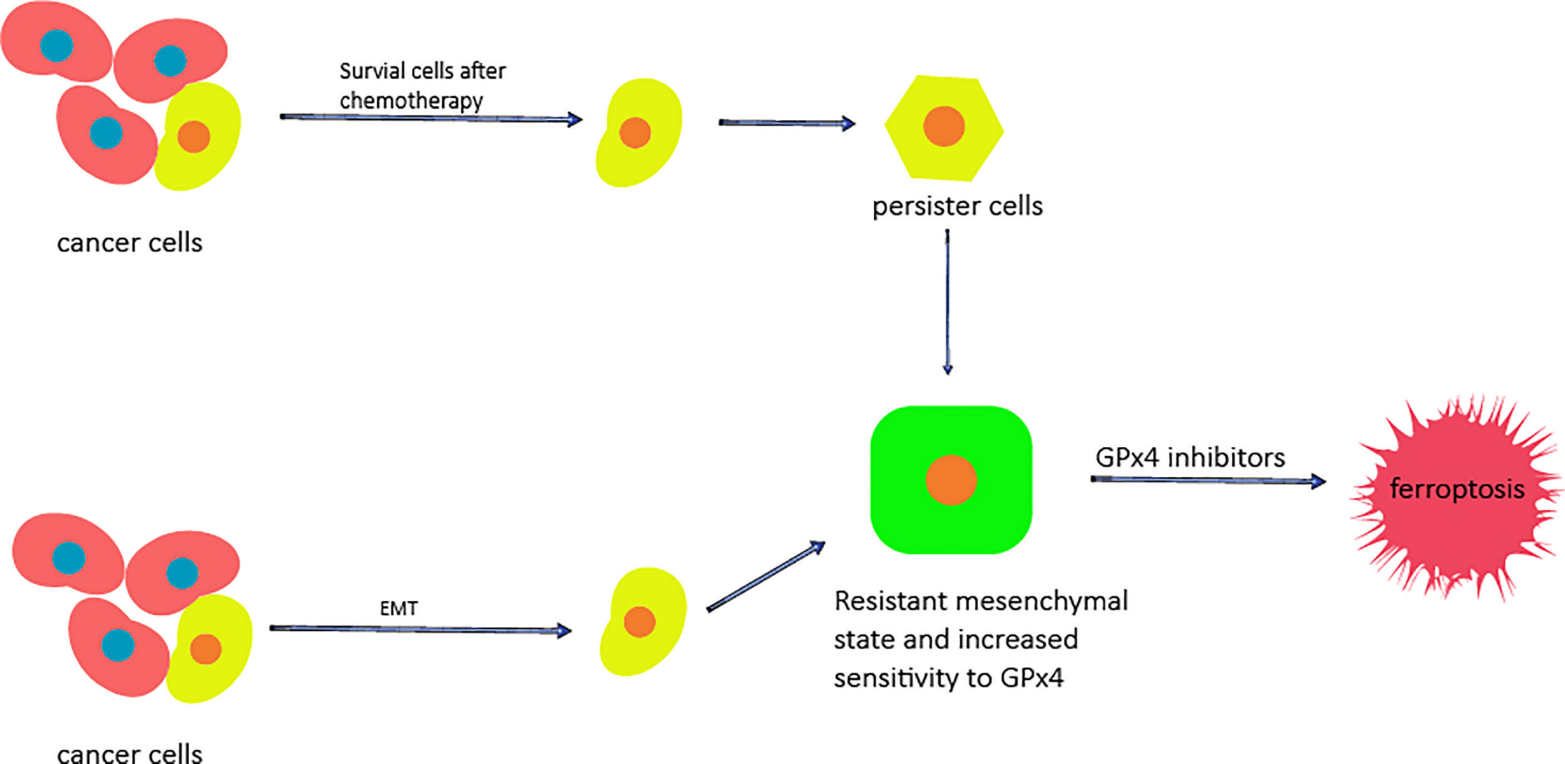
The potential of ferroptosis for overcoming cancer cell resistance. The cells that survive after chemotherapy are called persister cells and have mesenchymal characteristics. Mesenchymal markers and stem cell markers are increased, while the levels of GSH, NADPH and Nrf2 are decreased. Cancer cells can enter the mesenchymal state through EMT. In the mesenchymal state, the sensitivity of cells to GPX4 is increased, and the use of GPx4 inhibitors can cause them to die.

The resistance of cancer cells to chemotherapy is also a complicated problem in cancer treatment and results in most chemotherapy drugs failing to induce the death of cancer cells. Since ferroptosis is a cell death process completely different from apoptosis, the use of ferroptosis inducers may be a promising strategy for overcoming the lack of cell death induce by chemotherapeutic drugs (57). Specifically, epithelial cancer cells enter the stromal state through epithelial-mesenchymal transition (EMT), which is a process enabling cells to resist cell death through a variety of mechanisms, including inactivation of a large number of cancer cell apoptosic processes (57–59). Determining the vulnerability of these mesenchymal cancer cells is a promising strategy for improving treatment methods. Some studies have found that cancer cells in the mesenchymal state have higher enzyme activity than those in the epithelial state, which promotes the synthesis, use and storage of long-chain PUFAs. Long-chain PUFAs are an important source for reactive lipid peroxidation, which makes mesenchymal cancer cells highly dependent on GPx4 and sensitive to the inhibition of GPx4 (53); in addtion, cancer cells in the mesenchymal state also show a high level of iron compound sensitivity (57). Therefore, ferroptosis inducers can be used to promote the death of drug-resistant cancer cells in the mesenchymal state (**Figure 2**). Ferroptosis can also overcome the resistance of cancer cells by inducing the death of persister cells (**Figure 2**); persister cells are cancer cells that survive after several rounds of chemotherapy, and persister cell represent another treatment-resistant cell type in a variety of tumors (60, 61). Using persister cancer cells as a therapeutic target is also an important strategy for overcoming the drug resistance of cancer cells. Stem cell markers and mesenchymal markers are upregulated in persister cells, indicating the mesenchymal status of these cancer cells (62). Studies on the vulnerability of persister cancer cells revealed that the Nrf2 target gene is downregulated. Nrf2 is the main factor inhibiting of ferroptosis. Further studies showed that the GSH and NADPH levels of persister cells were significantly reduced as a specific result of lipid peroxidation rather than general sensitivity to oxidative stress. In addition, it has been proven that GPx4 inhibitors play a specific lethal role in persister cells through ferroptosis (53, 57, 62); therefore, the induction of ferroptosis is a promising method for overcoming the drug resistance of cancer cells.

At the same time, studies have shown that ferroptosis can promote tumor growth by driving macrophages in TME (63). Oxidative stress-induced KRAS-mutated KRAS^G12D^ protein is released into the TME from cancer cells that die of autophagy-dependent ferroptosis. Macrophages then take up KRAS^G12D^, which is packaged into exosomes outside the cell, *via* AGER mediated uptake. Finally, KRAS^G12D^ induces macrophages to change to the M2 phenotype to promote cancer development.

#### Effect of RT in cancer

**Figure 3:**
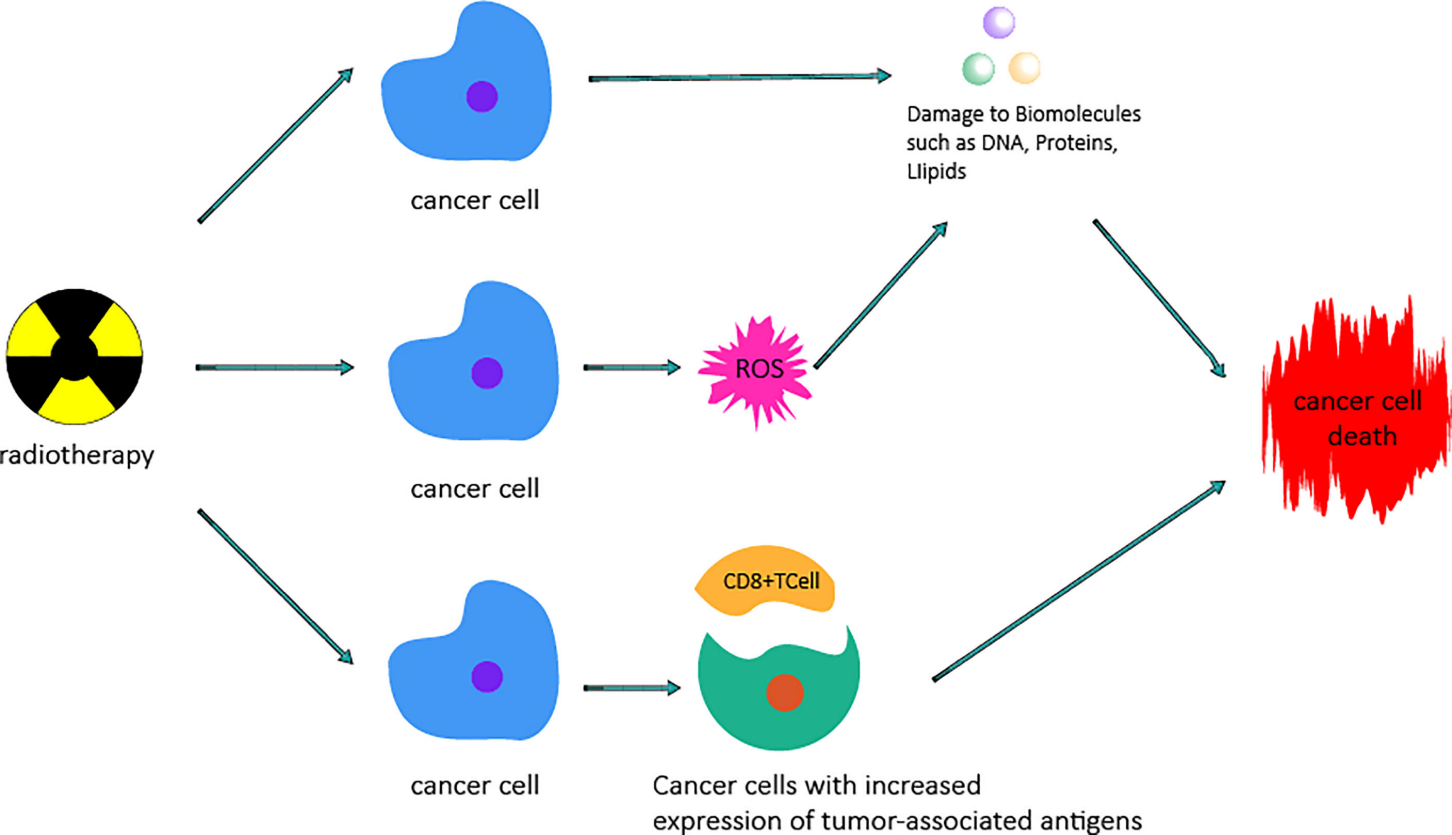
Mechanism by which radiotherapy kills cancer cells. Radiotherapy can cause damage to biomacromolecules such as DNA, protein, and lipids by direct actions or the generation of ROS by indirect actions, which can cause damage to biomacromolecules and then lead to cancer cell death. Radiotherapy increases the expression of tumor-associated antigens by altering the tumor microenvironment and stimulating the proliferation and priming of cytotoxic CD8+ T cells, thus causing cancer cell death.

Cancer is the leading cause of death worldwide. In 2012, the International Agency for Research on Cancer (IARC) reported 14.1 million new cancer cases, and 8.2 million of these cases result in death from cancer (64). Therefore, it is very important to find a safe and effective treatment for cancer. Roentgen discovered X-rays in 1895, and they were first used clinically in 1896 (65). In 1898, Marie Curie discovered radium and used radiation and X-rays in medical applications; radiation can kill cancer cells directly and cause DNA damage, leading to tumor cell death (66). Great progress has been made in the field of RT (67).In particular, the technical advances that have enable accurate application of radiation have revolutionized clinical RT, which has rapidly resulted in better local control and improved clinical results. In addition, the new understanding of radiobiology has transformed the treatment paradigm into one including biological accuracy and physical targeting to achieve individualized cancer treatment (68). RT is one of the main treatment methods currently in use. More than 50% of all cancer patients receive combined chemotherapy and surgery to treat various cancers (69). RT can be used not only for treatment but also for adjuvant therapy, depending on a variety of factors, especially the radiosensitivity of the tumor (70). Some cancers, such as laryngeal cancer, most lymphomas, cervical cancer and prostate cancer, as well as some types of central nervous system cancers, can be cured by RT (70). For palliative purposes, RT can not only be used to reduce anatomically unresectable tumors, such as those near key organs, blood vessels or the central nervous system, but can also be used to eliminate or relieve the pain caused by bone and brain metastasis of tumors and compression of the spinal cord (71–74). Irradiation(IR) usually refers to high-energy photon radiation, such as X-ray and gamma-ray radiation, as well as particle radiation, including α and β particles, carbon ions, electrons (E), protons and neutron beams (75, 76). RT is a treatment method using IR that can be used to achieve therapeutic objectives to meet different clinical needs. The basic principle of RT (**Figure 3**) is the interaction between IR and tumor cells. IR can cause effects by direct or indirect actions. In the case of direct actions, IR damages biological molecules, such as lipids and proteins, especially DNA, which is the most important effector of IR; DNA damage leads to the termination of cell division and proliferation and even leads to cell apoptosis or necrosis. Indirect actions destroy biomolecules through free radicals, mainly through ROS. ROS have unpaired electrons and can damage biomolecules through chemical reactions such as hydrogen extraction, addition, disproportionation and electron capture. These reactions can cause structural damage to biomolecules, such as single-or double-strand breaks of DNA and DNA-protein or DNA-DNA crosslinking, leading to cell death (77–79). ROS play a key role in RT, destroying biomolecules and activating relevant signaling pathways to promote apoptosis of tumor cells (78, 80–83). At the same time, studies have found that RT not only kills tumor cells directly but also changes the tumor microenvironment and enhances the immune system’s ability to recognize tumor cells, thus acting as a vaccine. RT leads to the release of cytokines, stimulates the recruitment of dendritic cells and, most importantly, stimulates the proliferation and activation of cytotoxic CD8+ T cells in the tumor microenvironment by increasing the expression of tumor-associated antigens. This immune cascade specifically generates activated T cells capable of inducing immunogenic cell death of cancer cells carrying such tumor-associated antigens (84). To minimize normal tissue exposure while also providing the maximum radiation dose to tumor cells, some improved strategies have been proposed in recent years, such as intensity-modulated RT (85–87), image-guided radiotherapy (IGRT) (88, 89), brachytherapy (90, 91), and stereotactic RT (92, 93). In addition, fractionated treatment regimens have been developed according to the principles of 5RS (reoxygenation, repair, redistribution, refill and intrinsic radiosensitivity) to enhance therapeutic efficacy (78, 94–96). Among the new strategies, IGRT can determine the location of tumors by guiding radiation beams, providing cancer tissues with as much radiation dose as possible while minimizing damage to key components caused by exposure to bystander cells (97). Stereotactic systemic radiotherapy (SBRT) is another relatively new method for ablating well-defined small tumors such as early non-small-cell lung tumors (98). This type of treatment employs a low fractionated dose regimen of less than five doses and has been shown to significantly improve local control and survival (99). By giving fewer, more precise, and higher doses of radiation, providers can provide treatment in a shorter time while retaining most of the normal tissue (100). In addition, RT alone or combined with other treatment methods can significantly improve the tumor cure rate. To prove this, researchers conducted a study on gastric cancer patients (97) and found that RT combined with chemotherapy provided a higher survival rate than chemotherapy alone. The average survival rate was 20.9 months for patients who received only chemotherapy after surgery and 46.7 months for patients who received chemoradiotherapy after surgery. Further illustrating the impact of radiation, 46.9% of patients who received chemoradiotherapy plus surgery survived five years after treatment, while only 24.9% of patients with chemotherapy plus surgery survived five years after treatment (97, 100). Therefore, RT has good therapeutic prospects in the treatment of tumors and is an indispensable tool for the treatment of tumors.

#### Combined use of ferroptosis and RT in cancer

RT is the cornerstone of many cancer treatments (101, 102), but radiation resistance is still the main cause of RT failure. Studies have found that RT can cause ferroptosis in tumor cells, and ferroptosis agonists can enhance the radiation efficacy of tumor models. To prove this, researchers used low-dose FINs, including sulfasalazine, RSL-3, erastin and atorvastatin, to pretreat HT1080, B16F10 and ID8 cell and then irradiated these cells. Compared with RT alone, FINs plus IR decreased the survival rate of HT1080 cells *in vitro* (103). In another study, HeLa and NCI-H1975 adenocarcinoma cells were irradiated and treated with erastin and/or X-rays, and cell clone formation ability, GPx4 expression, and GSH concentration were measured. *In vivo*, NCI-H1975 cells were transplanted into the left shoulder of nude mice, and the ability of erastin and GSH to induce radiosensitization was determined. After treatment with erastin, ferroptosis was observed in tumor cells, and the GSH concentration and GPx4 protein expression were decreased. In addition, erastin enhanced the cell death induced by X-ray IR in two human tumor cell lines. Similarly, *in vitro* studies, erastin treatment of transplanted tumor mice showed similar radiosensitization effects and reduced GSH concentrations in the tumor (104). The results showed that ferroptosis induction can be used as a targeted strategy to improve the efficacy of RT. At the same time, a single dose of 20 Gy (21 times) was given to B16F10 tumors resistant to RT and RSL3. It was observed that ferroptosis resistant tumors were still resistant to RT even at a higher dose of RT (103), indicating that resistance to ferroptosis induced by the ablation dose of RT led to resistance to RT. In other words, ferroptosis antagonists can limit the effectiveness of radiation. RT suppresses SLC7A11, a member of solute carrier family 7 and factor in the glutamate cystine reverse transporter XC-, by activating the ataxia telangiectasia mutated gene, resulting in reduced cysteine uptake, reduced GSH synthesis, enhanced tumor lipid oxidation and ferroptosis, and improved tumor control (103). Radiation can also promote lipid peroxidation, resulting in ferroptosis of tumors. RT can cause oxidative damage to all cell compartments, including the lipid membrane (105, 106). *In vitro* and *in vivo*, RT can increase the sensitivity of tumor cells to ferroptosis agonists, thus providing a new strategy for tumor radiosensitization. IR can not only induce ROS, but also induce the expression of ACSL4 (ACSL4 is a lipid metabolic enzyme that is essential for and the biosynthesis of PUFA- containing phospholipids, which are particularly vulnerable to peroxidation (107–109)), leading to increased lipid peroxidation and ferroptosis (110). ACSL4 ablation can largely eliminate the ferroptosis induced by IR and promote radiation resistance. Inactivation of SLC7A11 or GPx4 with FINs can sensitize radiation-resistant cancer cells and xenografts to IR. For example, sulfasalazine alone has a poor effect in inducing lipid peroxidation and inhibiting tumor growth, but it may enhance the sensitivity of tumors to IR through synergistic effects with IR-induced lipid peroxidation and ferroptosis (110). Sulfasalazine is a drug approved by the U.S. Food and Drug Administration (FDA) and is commonly used to treat rheumatoid arthritis; it has previously been identified as an inhibitor of SLC7A11 transporter activity (111). Therefore, the combination of sulfasalazine and RT is a promising treatment strategy in clinical practice. The expression of GPx4 is increased in radiation-resistant NSCLC cells. Downregulation of GPx4 or the use of a FIN targeting GPx4 improves the sensitivity of radiation-resistant lung cancer cells after high-dose and low fractionation IR. The expression level of SLC7A11 in biopsy tissues and glioma cell lines of patients with glioblastoma was higher than that in normal brain tissues (112); therefore, inhibition of system XC- or the GPx4 system by a FIN can enhance the RT effect in sarcoma, breast cancer and glioblastoma (113). Therefore, the combination of ferroptosis and RT can eradicate cancer cells resistant to conventional RT; the combination therapy shows more effective cancer cell lethality than monotherapy, and provides a promising method for the clinical treatment of cancer.

Radiation-induced pulmonary fibrosis (RILF) and radiation-induced lung injury (RILI) are life-threatening complications of thoracic radiotherapy. Studies (114) have shown that the use of ferroptosis inducers to induce ferroptosis in RILF mice will lead to increased levels of ROS, HYP and serum inflammatory cytokines in the lung of mice, thereby aggravating RILF. Some studies have also found that the use of ferroptosis inducers in mice with acute RILI led to the increase of ROS and inflammatory cytokines in the lungs of mice, which aggravated RILI. Decreased levels of GPx4 were also observed in RILI mice (115). Therefore, the combination of ferroptosis and RT may aggravate the damage of normal tissues such as lungs

## Discussion and perspective

In the treatment of cancer, in addition to inducing apoptosis and necrosis, ferroptosis can also be induced. Ferroptosis is a recently discovered regulatory form of cell death. Elucidation of its regulatory pathway will help to design FINs for targeted tumor therapy, and these can be combined with traditional tumor treatment methods. In this paper, we systematically reviewed the regulatory pathways of ferroptosis and revealed its potential in cancer treatment. We also noted that RT can not only restrict GSH synthesis by activating the ataxia-telangiectasia mutated gene to inhibit SLC7A11 of glutamate-cystine antiporter Xc- but also induce ferroptosis by inducing the expression of ROS and ACSL4. Moreover, the use of FINs can overcome the radiation resistance and acquired drug resistance of tumors and make tumors sensitive to RT again. Therefore, the combined use of ferroptosis and RT can not only overcome insensitivity to traditional treatment but also improve the killing tumor cells through synergistic effects; therefore, in cancer treatment, the combined application of ferroptosis and RT has broad therapeutic prospects.

However, due to the complexity of the biological system and the difficulties with clinical translation, the mechanism underlying the effects of ferroptosis combined with RT and whether the combination will induce uncontrollable consequences are not yet well understood. Successes *in vitro* experiments or animal models cannot be directly applied to clinical practice. Therefore, further investigation of the potential mechanism of the interaction between ferroptosis and RT will help the clinical application of combination therapy and avoid uncontrollable consequences. More importantly, ferroptosis is a double-edged sword, that can not only have a synergistic effect and improve the sensitivity of RT but also promote the occurrence and development of cancer. At the same time, some studies have found that ferroptosis may also be involved in RT-induced normal tissue damage, such as RT-induced lung injury (114, 115). Therefore, it is necessary to further study whether ferroptosis combined with RT is more toxic to tumor cells than normal tissues. Meanwhile, we should pay attention to the dose, duration and tissue specificity of FINs to avoid off-target toxicity in normal cells and tissues, and further basic and clinical studies are needed.

## Author contributions

ZL carried out the primary literature search, drafted and revised the manuscript, and participated in discussions. BX, WC, TT, QZ and XC helped modify the manuscript. All authors contributed to the article and approved the submitted version.
